# Analysis of the Pyrolysis Kinetics, Reaction Mechanisms, and By-Products of Rice Husk and Rice Straw via TG-FTIR and Py-GC/MS

**DOI:** 10.3390/molecules30010010

**Published:** 2024-12-24

**Authors:** Li Lin, Yang E, Qiang Sun, Yixuan Chen, Wanning Dai, Zhengrong Bao, Weisheng Niu, Jun Meng

**Affiliations:** 1 Biochar Engineering & Technology Research Center of Liaoning Province, College of Agronomy, Shenyang Agricultural University, Shenyang 110866, China; 2019200063@stu.syau.edu.cn (L.L.); eyang@syau.edu.cn (Y.E.); sunq33@outlook.com (Q.S.); 2 State Key Laboratory for Managing Biotic and Chemical Threats to the Quality and Safety of Agro-Products, Key Laboratory of Biotechnology in Plant Protection of Ministry of Agriculture and Zhejiang Province, Institute of Plant Virology, Ningbo University, Ningbo 315211, China; chenyixuan4161@163.com; 3 School of Agriculture, Liaodong University, Dandong 118001, China; ldxydaiwanning@163.com (W.D.); 18240129079@163.com (Z.B.); 4 College of Engineering, Shenyang Agricultural University, Shenyang 110866, China

**Keywords:** rice residues, pyrolysis, kinetics, master plot method, TG-FTIR, Py-GC/MS

## Abstract

This study employed thermogravimetric analysis (TGA), Fourier transform infrared spectroscopy (FTIR), and pyrolysis gas chromatography/mass spectrometry (Py-GC/MS) to characterize and provide insights into the pyrolysis behaviors and by-products of rice husk (RH) and rice straw (RS). The primary pyrolysis range is partitioned into three stages, designated as pseudo-hemicellulose, pseudo-cellulose, and pseudo-lignin pyrolysis, by an asymmetric bi-Gaussian function. The average activation energies of the three pseudo-components of RH were estimated by the Flynn–Wall–Ozawa and Starink methods to be 179.1 kJ/mol, 187.4 kJ/mol, and 239.3 kJ/mol, respectively. The corresponding values for RS were 171.8 kJ/mol, 185.8 kJ/mol, and 203.2 kJ/mol. The results of the model-fitting method indicated that the diffusion model is the most appropriate for describing the pseudo-hemicellulose reaction. The reaction of pseudo-cellulose and pseudo-lignin is most accurately described by a nucleation mechanism. An accelerated heating rate resulted in enhanced pyrolysis performance, with RS exhibiting superior performance to that of RH. RH produces 107 condensable pyrolysis by-products, with ketones, acids, and phenols representing the largest proportion; RS produces 135 species, with ketones, phenols, and alcohols as the main condensable by-products. These high-value added by-products have the potential to be utilized in a variety of applications within the agricultural, bioenergy, and chemical industries.

## 1. Introduction

The demand for energy has significantly increased due to rapid industrialization and steady population growth worldwide, resulting in the uncontrolled consumption of fossil fuels [1]. Nevertheless, the excessive utilization and consumption of petroleum have resulted in a multitude of environmental issues, including climate change and air pollution. The replacement of fossil fuel energy with renewable energy is an inevitable trend in the fight against global warming and the energy crisis. The utilization of biomass is considered a renewable and environmentally sustainable method to produce a wide range of chemicals and materials. It has been estimated that the total biomass supply from agriculture and forestry worldwide amounts to approximately 11.9 Bt dry matter per year, with 61% coming from agriculture [2]. It is projected that bioenergy will account for a significant portion of the world’s energy consumption by 2050, with estimates ranging from 35 to 65 percent [3].

The wide geographic distribution, renewable nature, low carbon footprint, and low SO_x_ and NO_x_ emissions associated with crop residues contribute to their promise as a biomass feedstock for multiple uses, such as bioenergy, by-product utilization, and emission reduction [4]. Rice is one of the most significant food crops worldwide, with a total cultivated area of 165 million hectares and a biological yield of 776 million metric tons in 2022 [5]. In view of their extensive cultivation area and high biomass content, rice residues are an important potential source of biomass energy that could reduce the heavy dependence on fossil fuels [6,7]. Statistical data indicate that China’s annual production of rice straw exceeds 200 million tons [8], and according to the calculation that rice husk accounts for 20% of rice straw, the amount of rice husk produced is more than 40 million tons. Despite the high bioenergy potential of rice residues, open burning, which produces air pollution, is still the main method of disposal of rice residues, and this measure is prohibited by the Chinese government. Therefore, effective utilization of these large quantities of rice residues is essential.

Pyrolysis is an invaluable thermochemical technique that offers a promising “waste-to-energy” solution, whereby the output can be precisely tailored to produce an extensive range of alternative products aligned with the specific requirements of the intended end user [9]. Pyrolysis converts biomass under anaerobic or oxygen-limited conditions into green biothermal energy and bioactive chemicals, including biochar, bio-oils and gases [10,11,12]. It would appear that there is a paucity of data pertaining to the pyrolysis characteristics of rice residues. This is a significant gap in the available information given the importance of understanding the pyrolysis behaviors and mechanisms of rice biomass materials. The pyrolysis products and reaction mechanisms of RH and RS have been previously investigated by numerous researchers, respectively [13,14,15]. However, these studies lacked an in-depth evaluation of the reaction mechanisms as well as analyses of rice residue pyrolysis based on deconvolution and statistical calculations. Fully understanding the chemical processes involved in biomass pyrolysis is vital for predicting the relevant reactions, which in turn inform the optimal design and operation of reactors. Moreover, the properties and reaction mechanisms of pyrolysis by-products remain to be investigated.

The pyrolysis of biomass involves complex thermochemical reactions that can be explained via various reaction models, including models related to nucleation, diffusion, and contraction [16,17]. Clarification of the reaction mechanisms and kinetics is critical in advancing our understanding of the dynamic transformation of biomass and its three principal components: hemicellulose, cellulose, and lignin. Model-free and fitting methods allow information to be obtained from thermogravimetric (TG) curves, including the reaction mechanism and the associated kinetic parameters (reaction mechanism function *f*(*α*), activation energy *E_a_*, and preexponential factor *A*, collectively known as the kinetic triplet). Model-free approaches such as the Friedman, Flynn–Wall–Ozawa, Kissinger–Akahira–Sunose, and Starink methods can be used to estimate the value of *E_a_* without identifying the reaction mechanism [18,19,20]. In a recent study, Nisar et al. [21] estimated the reaction kinetics of biomass pyrolysis, revealing the underlying reaction mechanism and the simplicity of the pyrolysis process. Scientists have combined model-fitting methods for master plots with model-free approaches to select the most appropriate model for the reaction mechanism [22,23].

Elucidating the volatile products and gases that are generated during the pyrolysis of biomass and evaluating the potential environmental consequences of this process are highly important. Fourier transform infrared spectroscopy (FTIR) has been employed for real-time monitoring of evolved gases and characterization of their functional groups [24]. Pyrolysis gas chromatography/mass spectrometry (Py-GC/MS) further facilitates the identification of derivatives resulting from rapid biomass pyrolysis, eliminating the need for subsequent reactions [25]. Due to the complexity of the pyrolysis reaction, the combination of Py-GC/MS with FTIR enables more accurate identification of the gaseous products released during pyrolysis [26]. Nevertheless, little attention has focused on the pyrolysis of rice residues through both Py-GC/MS and TG-FTIR, particularly a comparative by-products analysis of the properties generated by RH and RS pyrolysis. A significant knowledge gap exists in the literature concerning the bioenergy potential, reaction kinetics, and mechanisms of rice residue pyrolysis. The aforementioned challenges can be addressed to facilitate the design, scaling up, and optimization of pyrolysis applications, which will in turn enable the generation of more bioenergy from agricultural waste, as well as high-value-added products, promoting environmental and energy security. Thus, this study aimed to quantify and compare the thermal decomposition behavior, kinetic mechanisms, and gaseous by-products of RH and RS by combining FTIR and Py-GC/MS analyses.

## 2. Results and Discussion

### 2.1. Biopyrolysis Potential of RH and RS

In order to elucidate the properties of the pyrolysis of RH and RS, an analysis was first conducted on the elements and composition of these residues. As illustrated in Table 1, the nitrogen contents of RH (0.47%) and RS (0.64%) are less than that of waste dahlia flowers (3.66%) and tobacco straw (0.98%) [27,28], and the sulfur contents are considerably lower than that of beech (0.70%) [29]. RH and RS are more environmentally friendly in terms of the potential for nitrogen oxide and sulfur oxide emissions during pyrolysis. Both materials have a high volatile matter content, low ash conversion, and acceptable bioenergy recovery. The lignin content in RS (14.05%) is lower than that in RH (21.21%), indicating that RS is more appropriate for conversion to gaseous and liquid products. The HHV value of RS (15.69 MJ/kg) is higher than that of RH (14.16 MJ/kg), which suggests that RS has a greater energy release potential.

The inherent properties of different biomasses are distinct, exhibiting a variation in chemical content across their main functional groups. The FTIR spectra of the RH and RS raw materials are provided herewith for reference. Figure S1 illustrates the Fourier transform infrared (FTIR) spectra of the two biomass raw materials, RH and RS. The broad peak observed in the range of 3600 to 3000 cm^−1^ can be attributed to the stretching vibration of the OH groups present in cellulose, hemicellulose and lignin [30]. The region between 1800 and 800 cm^−1^, which may be considered the ‘fingerprint’ region, corresponds to the stretching vibration of various chemical groups present in lignocellulosic materials. The 1724 cm^−1^ peak corresponds to the C=O stretching vibration of the carbonyl and acetyl groups in xylan and the chemical groups present in lignin. The absorption peak of RH exhibits a markedly higher intensity than that of RS. The spectral band between 1540 and 1620 cm^−1^ is attributed to the C=O stretching vibrations. The band at approximately 900 cm^−1^ is indicative of the presence of sugars in cellulose and hemicellulose, and the RS exhibits a higher intensity at these two absorption peaks. The band at 1433 cm^−1^ is attributed to the -CH bending vibration of alkanes. The band at 1227 cm^−1^ represents the C-O stretching vibration in the cyclohexanol ring [31], and RS exhibits a notable trough in this region.

### 2.2. TG Characterization

#### 2.2.1. Thermal Decomposition Behavior

**Figure 1:**
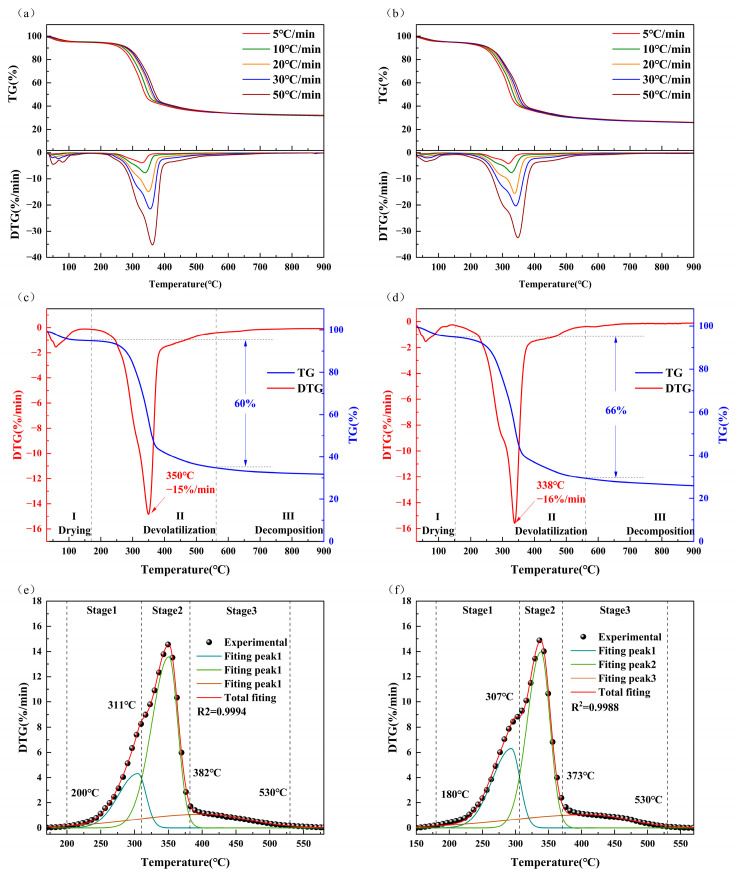
TG and DTG curves of biomass pyrolysis at five heating rates: (**a**) RH and (**b**) RS; TG and DTG curves of biomass pyrolysis at 20 °C/min: (**c**) RH and (**d**) RS; deconvolution of the devolatilization phase at 20 °C/min: (**e**) RH and (**f**) RS.

The pyrolysis behavior of rice residues was analyzed using a thermogravimetric analyzer at varying heating rates. As the rate of heating increased, a shift in the thermogravimetric curve toward the high-temperature range was observed (Figure 1a,b). This is evidenced by the DTG curves, which indicate that the temperature at the maximum rate of decomposition of RH rose from 329 °C to 362 °C and that RS exhibited a similar pattern. This suggests that more energy is required to achieve higher decomposition rates. Nevertheless, the heating rate had no discernible effect on the overall trend in the (D)TG curves, indicating that the pyrolysis reaction mechanism of RH and RS remained unaltered. Increasing the heating rate increased the number of decomposition reactions occurring within the biomass. These reactions resulted in a notable decrease in mass within a relatively brief timeframe. For simplicity, the TGA and DTG curves obtained under pyrolysis at 20 °C/min were selected for further analysis.

The pyrolysis of RH and RS was divided into the following three phases (Figure 1c,d): (1) the water loss phase (30~171.6 °C for RH and 30~152.8 °C for RS), in which the adsorbed water in the biomass evaporated with a low mass loss of 4.25% for RH and 4.57% for RS; (2) the degassing stage (171.6~562.5 °C for RH and 152.8~561 °C for RS), in which the RH mass loss was 60%, with a maximum rate of mass loss of 15%/min at 350 °C, and the RS mass loss was 66%, with a maximum rate of mass loss of 16%/min at 338 °C; and (3) the decomposition stage (562.5~900 °C for RH and 561.1~900 °C for RS), characterized by the gradual deterioration of residual lignin and carbon-based material, accompanied by a slight mass loss, with a final residual mass of 32% for RH and 26% for RS. This discrepancy is due to the lower (hemi)cellulose content and higher lignin content of RS than RH, in addition to the fact that RH contains a greater quantity of ash.

#### 2.2.2. Deconvolution of the Devolatilization Stage

The reaction kinetics of the three pseudo-components in each stage can be comprehensively understood by dividing the principal thermal degradation process (devolatilization stage) into three substages through the use of the peak fitting tool in Origin 2023. This methodology has been employed to analyze the thermal degradation of wastewater sludge, waste tea, and bamboo residue [22,23,32]. For illustrative purposes, consider the case where *β* = 20 °C/min. The dataset was divided into three stages by identifying the intersections of the three subpeaks (Figure 1e,f). The curves demonstrate a high level of fit (0.9994 for RH and 0.9988 for RS), indicating that the asymmetric bi-Gaussian model accurately describes the deconvolution process. For the first stage (pseudo-hemicellulose), the temperatures for RH were between 200 °C and 311 °C, and those for RS were between 180 °C and 307 °C. The observed reduction in weight was attributed primarily to the degradation of hemicellulose. Since hemicellulose consists of nonhomogeneous polysaccharides composed of different kinds of sugar molecules with a relatively loose structure and low degree of polymerization, it usually decomposes between 200 °C and 310 °C [33]. In the second stage (pseudo-cellulose), the temperatures for RH ranged from 311~382 °C, and those for RS ranged from 307~373 °C. The primary factor contributing to weight loss at this stage is the thermal decomposition of cellulose and minor quantities of lignin. Cellulose is a chain polymer composed of glucose molecules that is structurally ordered and stable, and it decomposes between 300 °C and 400 °C [34]. In the third stage (pseudo-lignin), the temperatures for RH ranged from 382 to 530 °C, and those for RS ranged from 373 to 530 °C. The weight loss observed during this stage is attributed primarily to the decomposition of lignin. Due to its complex polymeric composition, which comprises aromatic rings and side chains, lignin exhibits greater stability and chemical bond strength than hemicellulose. Consequently, it is more challenging to degrade, with a decomposition range of 170 °C to 900 °C [35]. The pyrolysis temperature intervals of three pseudo-components were all within the intervals for the corresponding components, demonstrating the reliability of the asymmetric bi-Gaussian model.

### 2.3. Kinetic Analysis

#### 2.3.1. *E_a_* Estimated by Model-Free Approaches

**Figure 2:**
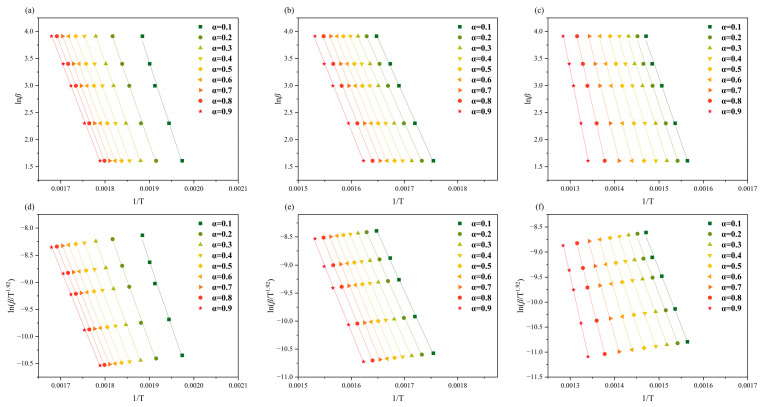
(**a**–**c**) FWO plot and (**d**–**f**) Starink plot for the three subphases of RH.

**Figure 3:**
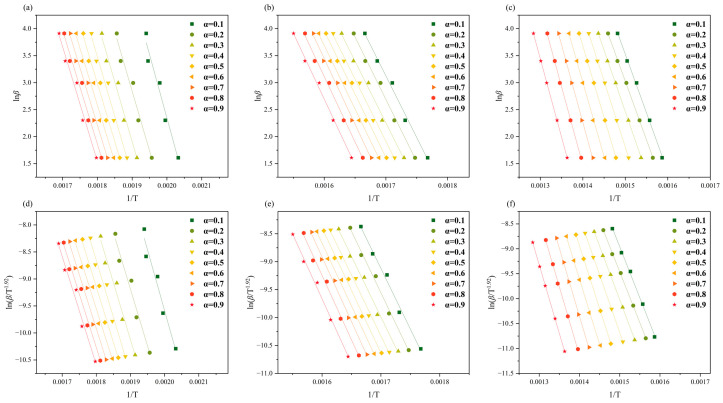
(**a**–**c**) FWO plot and (**d**–**f**) Starink plot for the three subphases of RS.

*E_a_* was estimated via two methods, the FWO and Starink methods, for each of the three substages of the devolatilization phase of RH and RS, where α ranged from 0.1 to 0.9 with increments of 0.1. As shown in Figure 2 and Figure 3, *lnβ* (FWO) and *ln*(*β*/*T*^1.92^) (Starink) versus 1/*T* showed a linear relationship at the same conversion rate. *E_a_* was estimated from the gradient of the regression line, and the coefficient of determination (*R*^2^) ranged from 0.9475 to 0.9999 (Table 2).

The average *E_a_* values obtained by the FWO method across the three substages of RH thermal degradation were 179.3, 187.6, and 239.9 kJ/mol, which exhibited a slight increase relative to the average *E_a_* values obtained by the Starink method (178.8, 187.2, and 238.7 kJ/mol, respectively). Similarly, the average *E_a_* values estimated by the FWO method for the three substages of RS thermal degradation were slightly higher than those estimated via the Starink method. However, the *E_a_* values estimated by the two methods were very close to each other, so their average values were used in the following analyses. The estimated values for RH were 179.1, 187.4, and 239.3 kJ/mol, whereas those for RS were 171.8, 185.8, and 203.2 kJ/mol. The *E_a_* values of the three substages of RH and RS are ordered from largest to smallest as pseudo-lignin, pseudo-cellulose, and pseudo-hemicellulose. This result is attributable to the thermally labile nature of hemicellulose, which is less stable than cellulose and parts of lignin [25]. The *E_a_* values of all three pseudo-components of RH are greater than those of the pseudo-components of RS, indicating that RS is more susceptible to cleavage than RH. A lower activation energy indicates that the biomass is more susceptible to thermochemical conversion. Consequently, RH and RS should be designed with different thermo-chemical conversion processes when they are used as raw materials to produce bioenergy or biochemicals.

#### 2.3.2. Determination of the Reaction Mechanism

To comprehensively understand the kinetic reaction mechanism, it is essential to accurately estimate the pre-exponential factor (*A*), the order (*n*), and the reaction model. Once the value of *E_a_* has been determined, a kinetic model can be generated through the integral master plot method. The theoretical master plot is then compared with the experimental master plot to determine the most appropriate model. On the basis of the previously determined value of *E_a_* and the measured temperature as a function of conversion (*α*), the value of *p*(*x*) can be calculated from Equation (9). The relationship between *p*(*x*)/*p*(*x*_0.5_) and *α* is illustrated in Figure S2. The *p*(*x*)/*p*(*x*_0.5_) curves of each substage are essentially identical for the five heating conditions. This result indicates that the pyrolysis kinetic mechanism remains consistent with changes in heating rate, suggesting that a single model can adequately describe the behavior.

**Figure 4:**
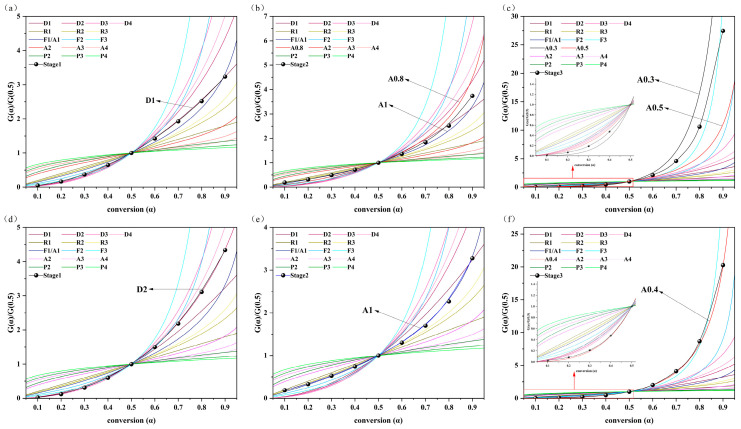
Comparison of *G*(*α*)/*G*(0.5) versus conversion for the three substages of (**a**–**c**) RH and (**d**–**f**) RS with reaction modeling at 20 °C/min.

The theoretical *G*(*α*)/*G*(0.5) curves of individual substages were compared with the corresponding experimental *p*(*x*)/*p*(*x*_0.5_) curves at *β* = 20 °C/min (Figure 4). The *G*(*α*)/*G*(0.5) of the pseudo-hemicellulose stage for RH is approximately aligned with the theoretical master plot D1 (*R*^2^ = 0.9996); the pseudo-cellulose stage is between A0.8 and A1, and the pseudo-lignin stage is between A0.3 and A0.5. The pseudo-hemicellulose stage and the *G*(*α*)/*G*(0.5) of the pseudo-cellulose stage for RS were near D2 and A1, and the pseudo-lignin stage is near A0.4.

#### 2.3.3. Determination of Reaction Order and Preexponential Factor

The thermal degradation stages of pseudo-cellulose and pseudo-lignin for RH and pseudo-lignin for RS were consistent with the A*n* model. To further refine the reaction mechanism, the reaction model of *G*(*α*) = [−ln(1 − *α*)]^1/*n*^ was inserted into Equation (6) to obtain Equation (1):

(1) $$G\left(\alpha \right)=\frac{AE}{\beta R}P\left(x\right)={\left[-\ln\left(1-\alpha \right)\right]}^{1/n}$$

**Figure 5:**
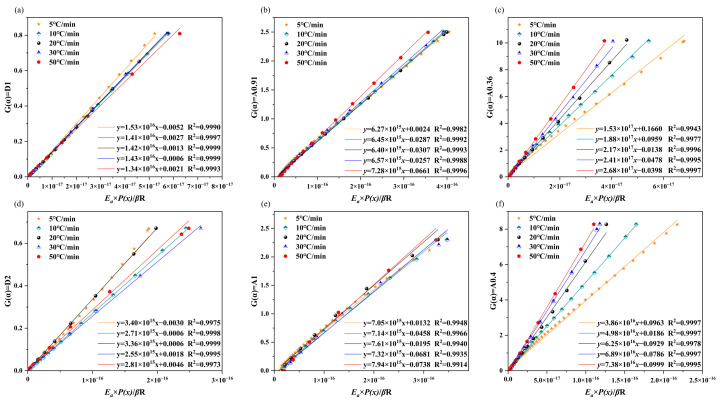
*G*(*α*) versus *EaP*(*x*)/*β*R for the three substages of biomass pyrolysis: RH (**a**–**c**) and RS (**d**–**f**).

To further verify the optimal value, a linear regression of *G*(*α*) with respect to $\frac{E_{a}p(x)}{\beta R}$ was fitted by the least squares method by increasing *n* from 0.1 to 1 in steps of 0.01 according to Equation (10). The best-fit value of *n* was estimated on the basis of the intercept closest to zero (Figure 5). The pseudo-cellulose and pseudo-lignin pyrolysis stages of RH corresponded to A0.91 (*f*(*α*) = 0.91(1 − *α*)[−ln(1 − *α*)]^−9/91^) and A0.36 (*f*(*α*) = 0.36(1 − *α*)[−ln(1 − *α*)]^−16/9^), respectively, and the pseudo-lignin stage of RS is consistent with A0.4 (*f*(*α*) = 0.4(1 − *α*)[−ln(1 − *α*)]^−3/2^). Similarly, the pyrolysis of hemicellulose has been demonstrated to be primarily regulated by a diffusion mechanism [36], and the pyrolysis reactions of cellulose and lignin are consistent with a nucleation model [23].

The kinetic triplets of RH and RS under the five heating rates are shown in Table 3. The magnitude of the preexponential factor can differentiate between the various categories and fundamental characteristics of chemical reactions that occur during pyrolysis [37]. When A is less than 1 × 10^9^ s^−1^, the simplest surface chemical reactions are assumed to occur; conversely, when A ≥ 1 × 10^9^ s^−1^, the chemical reaction pathways are assumed to be relatively complex. Therefore, for each pseudo-component of RH and RS with A values in the range of 2.55 × 10^15^~2.68 × 10^17^ s^−1^, the evaporation behavior is considered to involve a relatively simple and homogeneous chemical reaction.

**Figure 6:**
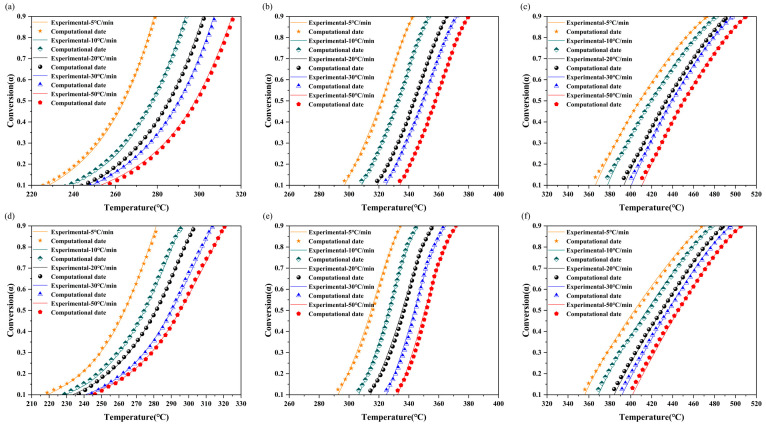
Comparison of the calculated values of the three substages of (**a**–**c**) RH and (**d**–**f**) RS with experimental data at five heating rates.

To validate the kinetic results, Figure 6 compares the theoretically derived model calculations with the experimental curves corresponding to each substage. The calculated kinetic parameters exhibit a high degree of correlation with the corresponding experimental data, thereby confirming the validity of the kinetic analysis results. The results of the present study provide support for the use of RH and RS in practical industrial applications, as well as for the design of their pyrolysis reactors.

### 2.4. Pyrolysis Performance

Table 4 lists the indicators related to the pyrolysis performance of RH and RS. *T_i_* and *T_p_* exhibited a positive linear correlation with the heating rate, and the values for RH exceeded those for RS at identical heating rates. This suggests that changes in heating rate have a greater influence on heat transfer in RH than in RS. The observed rise in *R_p_* and *R_e_* values with increasing heating rate indicates that elevated heating rates are conducive to enhanced pyrolysis, whereas the *CPI* (Comprehensive Pyrolysis Index) values demonstrate that an accelerated heating rate improves pyrolysis performance. The *CPI* of RS (4.98–246.83) was greater than that of RH (3.42–198.46) when the rate of heating was held constant, indicating that the pyrolysis performance of RS was superior to that of RH. In industrial production, the two most important operating factors are pyrolysis time and pyrolysis efficiency. A shorter reaction time and enhanced pyrolysis performance at a heating rate of 50 °C/min represent an optimal choice for the pyrolysis of rice residues.

### 2.5. TG-FTIR Analysis

**Figure 7:**
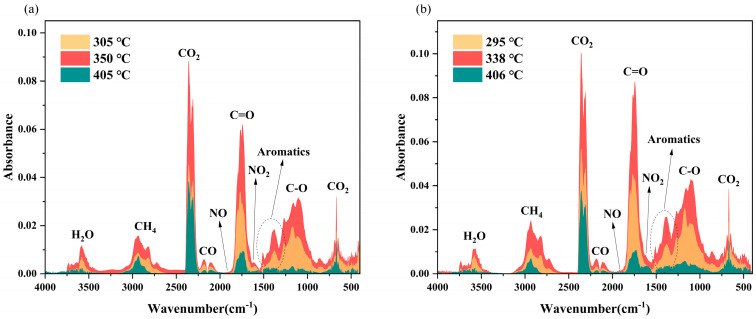
Infrared spectra of pseudo-component peaks: RH (**a**) and RS (**b**).

The macromolecules of (hemi)cellulose and lignin, which are the primary components of RH and RS, undergo a series of chemical transformations during pyrolysis. These reactions include depolymerization, cross-linking polymerization, dehydrogenation, and deoxygenation, which ultimately generate gaseous low-molecular-weight compounds [38]. Figure S3 shows the 3D FTIR spectra of RH and RS pyrolyzed at 20 °C/min. In the initial stage, the weight loss of both RH and RS was minimal, resulting in the absorbance values of the detected compounds in the 3D FTIR spectra being close to zero. The majority of the pyrolyzed volatiles were detected in the temperature range corresponding to the primary weight loss region. The evolved gases were identified on the basis of their absorbance at the maximum temperature of the three substages of the principal pyrolysis phase (Figure 7), with the greatest intensity of gas release observed at 350 °C and 338 °C for RH and RS, respectively. Table 5 lists the gas products of the two residues, along with the corresponding functional groups.

**Figure 8:**
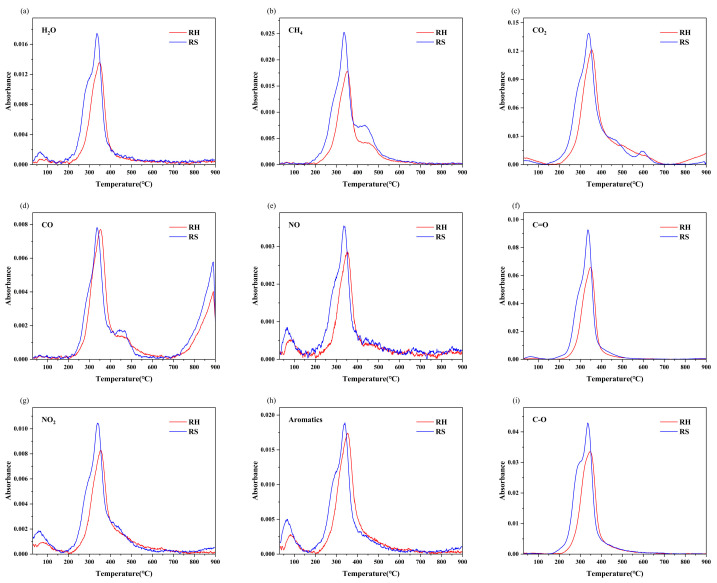
Gas emissions from RH and RS pyrolysis and their peak absorbance results. (**a**): H_2_O, (**b**): CH_4_, (**c**): CO_2_, (**d**): CO, (**e**): NO, (**f**): C=O, (**g**): NO_2_, (**h**): Aromatics, (**i**): C-O.

Figure 8 shows the infrared spectra of the principal pyrolysis products of RH and RS as a function of temperature. The absorption peaks within the 4000–3450 cm^−1^ range indicate the release of H_2_O. The whole pyrolysis process of RS was accompanied by the emission of H_2_O, but RH produced almost no H_2_O above 700 °C. The generation of H_2_O during low-temperature pyrolysis can be attributed to the release of three distinct types of water: bulk water, bound water, and crystallization water. As the temperature rises, oxygen-containing functional groups also decompose and react, contributing to the production of H_2_O [26]. The band from 3100 to 2650 cm^−1^ represents C–H stretching vibrations and is indicative of the release of CH_4_. Both RH and RS exhibit a pronounced CH_4_ release peak and a relatively weak secondary peak. However, the latter peak is more prominent in RS than in RH, occurring at temperatures between 400 °C and 500 °C. The emission of methane is attributed to the cleavage of methoxy (-O-CH_3_), methyl (-CH_3_), and methylene (-CH_2_-) groups from the side chains of hemicellulose and lignin. The distinctive peaks at 2400–2250 cm^−1^ and 750–600 cm^−1^ are indicative of CO_2_. The production of CO_2_ is attributed to the decomposition of carboxyl (-COOH), carbonyl (-C=O), and esteryl (-COOR) groups, in addition to the decomposition of C-C and C-O bonds in hemicellulose and cellulose side chains [39]. The release of CO_2_ from RS is relatively weak at 600 °C, which may be attributed to the secondary degradation of C=O. The absorption peak in the range of 2230–2020 cm^−1^ is attributed to CO emission. CO gas is generated primarily through the cleavage and subsequent recombination of ether bonds (C-O-C) and carbonyl groups (C=O). At elevated temperatures, the pyrolysis of residual char facilitates the reduction of CO_2_ to CO (CO_2_ + C = 2CO) [40]. Consequently, a notable increase in the intensity of CO release occurs above 700 °C. The two peaks at absorption wavelengths of 2000–1870 cm^−1^ and 1640–1570 cm^−1^ indicate the release of NO and NO_2_, respectively.

Organic compounds make up a large proportion of pyrolysis gases, and these gaseous compounds can be condensed into liquids, which can be used as energy sources, chemical feedstocks, or organic fertilizers. The peak at 1850–1610 cm^−1^ corresponds to the vibration of C=O, representing the release of acids, aldehydes, ketones, and esters, which are produced mainly by the cleavage of epoxy functional groups (-CH(O)CH-). The presence of aromatic compounds is indicated by the C–C backbone vibration peak within the range of 1610–1420 cm^−1^. Additionally, the stretching vibration peak at 1420–1000 cm^−1^ is indicative of hydroxyl compounds. The release of these organic compounds occurs mainly between 200 °C and 600 °C.

**Figure 9:**
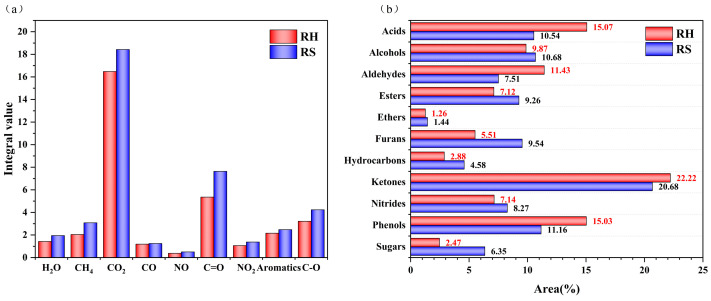
(**a**) Yields of gases from RH and RS pyrolysis; (**b**) relative yields of organic pyrolysis by-products from RH and RS.

Rombauer’s Law postulates a positive correlation between the concentration of a gas and its absorbance. The order of intensity of gas emissions is CO_2_ > C=O > C-O > aromatics > CH_4_ > H_2_O > CO ≈ NO_2_ > NO for RH and CO_2_ > C=O > C-O > CH_4_ > aromatics > H_2_O > NO_2_ > CO > NO for RS (Figure 9a). Both RH and RS produce the highest emissions of CO_2_ and the lowest emissions of CO and NO. The low concentrations of NO_2_, NO, and CO confirm that RH and RS are clean biomasses.

TG-FTIR analysis provided insights into the pyrolysis products and their corresponding functional groups. Both RH and RS exhibited characteristic release patterns of gases and organic compounds, with notable differences in peak intensities and composition. The observed emissions confirm the clean biomass nature of RH and RS and suggest that these materials have potential for applications in energy production and organic synthesis.

### 2.6. Py-GC/MS Analysis

Py-GC/MS analysis revealed a diverse array of compounds in the pyrolysis products of RH and RS, which were classified into distinct chemical groups. The observed differences in compound yields and composition reflect the distinct pyrolysis behaviors of RH and RS, which are influenced by their unique biomass compositions. The identified compounds offer significant insights into the prospects for the utilization of RH- and RS-derived products in a range of industrial sectors.

The pyrolysis products of RH and RS identified by Py-GC/MS are listed in Table S1. On the basis of comparison with the NIST spectral library, 107 and 135 compounds were identified for RH and RS, respectively. The identified compounds were categorized into 11 groups: acids, alcohols, aldehydes, esters, ethers, furans, hydrocarbons, ketones, nitrogens, phenols, and sugars. The main products of RH were ketones, acids, and phenols, which together accounted for 52.32% of the total emitted compounds (Figure 9b). The ketones included 2-propanone, 1-hydroxy- (5.60%), 1-hydroxy-2-butanone (2.72%), and 2,3-butanedione (2.55%). The phenols included 2-methoxy-4-vinylphenol (2.55%), phenol, 2-methoxy (2.50%), and 2,6-dimethoxy-phenol (2.09%). Acids included mainly acetic acid (12.08%) and n-hexadecanoic acid (2.46%). Ketones, phenols, and alcohols were the main pyrolysis products of RS, and these three types of compounds accounted for 42.52% of the total amount of emissions. The ketones included hydroxyacetone,2-propanone,1-hydroxy- (3.90%), cyclopentanedione 1,2-cyclopentanedione (3.04%), and acetone 1,2-cyclopentanedione, 3-methyl- (2.35%); phenols included 2-methoxy-4-vinylphenol (1.86%), 2,6-dimethoxy-phenol (1.74%) and phenol (1.35%); and alcohols included 1-propanol (3.66%) and 3-buten-2-ol (3.18%). The above C=O-containing compounds are the major pyrolysis products of RH and RS, which is supported by the TG-FTIR results.

In our study, the acid content of the RH pyrolysis by-products was 1.4 times higher than that of the RS by-products, and acetic acid was the compound with the highest pyrolysis yield for both materials. A previous investigation revealed that acetic acid is generated primarily by O-acetyl cleavage of hemicellulose polysaccharide units [41]. It is a commonly held belief that a high acid content will result in the destabilization of bio-oil, thereby reducing its quality. However, a high organic acid content in agricultural settings has been shown to yield superior outcomes [42]. Alcohols were derived mainly from the ring-opening reaction of the monosaccharides of hemicellulose; rapid pyrolysis of RS yielded 15 alcohols, while that of RH yielded 7, and the content of alcohols was higher in RS than in RH. Huang et al. [26] reported that the alcohols involved in the pyrolysis of water hyacinth consist of mainly oleyl alcohol, but 1-propanol was the most abundant alcohol in our study. Aldehydes are formed by β-bond cleavage of cellulose monomer molecules [43], and ketones are mainly formed by intramolecular ketonation after the dehydration of acids [44]; RH produced more aldehydes and ketones than did RS. Furans are formed mainly by dehydration of xylan units and levoglucan from cellulolytic decomposition [45], and the most prominent products among furans were 3-furanmethanol and 2(5H)-furanone. Prior research has demonstrated that the relative contents of furans produced by the pyrolysis of wheat and buckwheat are 5.46% and 0.47%, respectively. It is evident that the content of furans produced by the pyrolysis of rice residues in this study is markedly higher [46]. In this study, the sugar content was low among pyrolysis by-products; similarly, Zhang et al. reported a 3.06% sugar content in bamboo residue pyrolysis products [23]. β-d-Glucopyranose, 1,6-anhydro- was the highest-yielding sugar, with a content of 1.71% in RH and 4.50% in RS. Compared with that of RH, the rapid pyrolysis of RS resulted in a greater yield and variety of esters, mainly 2-hydroxy-gamma-butyrolactone and butyrolactone. In the early stages of pyrolysis, the amino acids are cleaved directly to HCNO, and as the temperature increases, the carbonyl and amino functional groups react to form nitrogen-containing compounds according to the Meladic reaction. The nitrogen compounds in RH include mainly 2(1H)-pyridine, and those in RS include mainly 1-propanol, 2-amino-, (S)-. Phenolic compounds are derived primarily from the degradation of lignin and can be produced in conjunction with cellulose decomposition intermediates through aromatization [47,48]. RH contains more lignin and thus produces more phenols than RS does. Lignin can be classified into three categories, syringyl, guaiacyl, and p-hydroxyphenyl, and fundamental units of lignin are evident among the phenolic compounds in Table S1. In general, the principal decomposition products of fast pyrolysis of RH and RS were comparable, although the relative yields of organic compounds exhibited notable differences.

The biomass pyrolysis gas is condensed into bio-oil, and the water-soluble substances in the lower layer can be extracted as wood vinegar or pyroligneous acid. The preceding studies have appraised the economic value of the technology utilized for the synthesis of bio-oil via fast pyrolysis, substantiating its prospective commercial viability [49]. Wood vinegar has been demonstrated to function as a plant growth regulator in agricultural applications [50]. Due to its high contents of phenols and ketones, it is also used as an insecticide and fungicide [51,52]. The alcohols in the pyrolysis products can be used as clean biofuels for the production of high-alcohol fuels, thus reducing environmental pollution. Hexadecanoic acid is a versatile acid used in food additives, cosmetics, and lubricants. Furfural is widely used in adhesives, organic solvents, and pesticides [53]. Butyrolactone is a common solvent and reaction reagent in chemistry. 2,6-Dimethoxyphenol has been shown to be a potent anti-inflammatory molecule [54]. Although the substantial quantities of by-products have considerable recovery value, the separation, purification, and enrichment of these materials for industrial applications represents a significant research topic. For example, ZnCl_2_ can be added to catalyze pyrolysis, thereby increasing the yield of aldehydes and ketones [55], and phenols can be isolated and purified by distillation [56]. It can be reasonably deduced that the pyrolysis by-products in question possess considerable economic value as energy sources and chemical raw materials.

In conclusion, the comprehensive characterization of the pyrolysis behavior of RH and RS provides valuable insights into their potential applications as sustainable biomass resources. The quality and composition of the products may vary depending on the biomass feedstock and the pyrolysis conditions. Further research should be conducted to develop additional biomass feedstocks and optimize the pyrolysis conditions in order to expand the industrial application and commercial value of pyrolysis technology.

## 3. Materials and Methods

### 3.1. Sample Preparation and Characterization

RH and RS samples were obtained from Shenyang Agricultural University. The samples were subjected to surface dust removal, subsequently dried at 105 °C until a constant weight was achieved, then ground and sieved to 200 mesh. The elemental composition of the samples, including the contents of C, H, N, and S, was determined using an elemental analyzer (Vario EL Cube). The O content was calculated by the difference method. The sample fractions were analyzed in accordance with the method of Van Soest, and the ultimate analysis, proximate analysis, and higher heating value (HHV) results are presented in Table 1. In order to carry out a comparative analysis of the functional group characteristics of the raw materials, Fourier transform infrared spectroscopy was used to characterize the raw materials in this study.

### 3.2. TG-FTIR Experiments

A thermogravimetric analyzer (Discovery TGA, TA Instruments, New Castle, PA, USA) was used to determine the nonisothermal pyrolysis behavior of the samples at five different heating rates (*β* = 5, 10, 20, 30, and 50 °C/min) in a nitrogen atmosphere. For each experiment, 10 ± 0.5 mg of sample was placed in a platinum crucible with the nitrogen flow rate set to 50 mL/min and heated from 30 to 900 °C. Prior to the experiments for each condition, preliminary experiments were conducted to eliminate any potential systematic errors in the system. To guarantee the replicability of the pyrolysis experiments, each experimental run was conducted a minimum of two times. To estimate the peaks of hemicellulose, cellulose, and lignin during pyrolysis, the absolute value of the second-order derivative (|DDTG|) was calculated for the TG curves [57]. For each of the three components, the temperature at which the absolute value of DDTG reaches a local minimum was considered the peak temperature. After the peak temperature was determined, the DTG curves were deconvoluted using an asymmetric bi-Gaussian function in Origin 2023 software.

The gases produced during pyrolysis at a rate of 20 °C/min were identified through FTIR (Thermo Fisher IS50, Thermo Fisher Scientific, Waltham, MA, USA). FTIR spectra were collected at a rate of eight scans per sample with a resolution of 4 cm^−1^ over the absorption wavelength range of 4000 to 400 cm^−1^. The gas from the TG pyrolyzer (TRIOS5.11, TA Instruments, New Castle, PA, USA) was transported into the FTIR gas chamber via a heated transfer line with N_2_ as the carrier gas. The transfer line temperature was heated to 300 °C prior to the experiment to avoid condensation and liquefaction of the precipitated gas. The experimental data were analyzed and processed via OMNIC software (OMNIC9, Thermo Fisher Scientific, Waltham, MA, USA) and TQ Analyst EZ Edition software (TQ Analyst EZ Edition, Thermo Fisher Scientific, Waltham, MA, USA).

### 3.3. Py-GC/MS Experiments

The volatiles resulting from pyrolysis were identified by Py-GC/MS. In the pyrolysis stage, a CDS5200 (CDS Analytical, Oxford, PA, USA) was used as the pyrolysis reactor, and 0.5 mg of sample was placed in a pyrolysis quartz boat and pyrolyzed at 600 °C for 15 s. The pyrolysis reactor was connected to a GC–MS (Agilent 7890B-5977A, Agilent Technologies Inc., Santa Clara, CA, USA) instrument with a 300 °C heating line, and the volatiles were separated in the GC section using a DB-1701 capillary column (60 m × 0.32 mm × 0.25 µm). Helium (99.999%) was employed as the carrier gas at a flow rate of 1 mL/min with a split ratio of 75:1. The column chamber temperature was maintained at 40 °C for one minute, after which it was increased at a rate of 5 °C/min to 280 °C, with a final hold of 10 min. The ion source temperature was 250 °C, the mass detection range (*m*/*z*) was 40–1000, 10 plots were scanned per second, the detection voltage was 1600 V, and the electron impact (EI) energy was 70 eV. The obtained mass spectra were compared with the NIST spectral library, and the relative contents of the compounds were calculated via normalization.

### 3.4. Kinetic Analysis

In pyrolysis reactions involving solid biomass, the relationship between conversion rate and temperature can be expressed according to the Arrhenius equation

(2) $$\frac{d\alpha }{dt}=A\;e^{-\left(\frac{E_{a}}{RT}\right)}f\left(\alpha \right)$$

In Equation (2), *A* represents the preexponential factor (s^−1^), also known as the frequency factor. *E_a_* denotes the activation energy (kJ/mol), *T* signifies the absolute temperature (K), *R* is the universal gas constant (8.314 J·K^−1^·mol^−1^), *f*(*α*) represents the reaction model, and *t* is the time (s). The conversion rate (*α*) is defined on the basis of the TG data as follows:

(3) $$\alpha =\frac{m_{0}-m_{t}}{m_{0}-m_{\infty }}$$

where *m*_0_, *m_t_*, and *m*_∞_ represent the initial, real-time, and final mass of the samples, respectively.

For experimental data obtained from nonisothermal experiments, Equation (2) can be modified via the following equation to obtain a nonisothermal expression for the rate of reaction as a function of temperature at a constant heating rate:

(4) $$\frac{d\alpha }{dT}=\frac{d\alpha }{dt}\cdot \frac{dt}{dT}$$

In Equation (4), the nonisothermal reaction rate is represented by *dα*/*dT*, the isothermal reaction rate by *dα*/*dt*, and the constant heating rate by *dT*/*dt*, which is typically denoted as *β*.

Upon substituting Equation (2) into Equation (4), we obtain the differential form of the nonisothermal rate equation at a constant heating rate:

(5) $$\frac{d\alpha }{dT}=\frac{A}{\beta }\;e^{-\left(\frac{E_{a}}{RT}\right)}f\left(\alpha \right)$$

Separating the variables and integrating Equations (2) and (5) yields the following integral forms of the nonisothermal rate equation:

(6) $$G\left(\alpha \right)=\int_{0}^{\alpha }\frac{d\alpha }{f\left(\alpha \right)}=\frac{A}{\beta }\int_{0}^{T}e^{-\frac{E}{RT}}dT=\frac{AE_{\alpha }}{\beta R}\int_{x}^{\infty }e^{-x}x^{-2}dx=\frac{{AE}_{\alpha }}{\beta R}\cdot p\left(x\right)$$

where $x=\frac{E_{\alpha }}{RT}$; *G*(*α*) is the integral form of the reaction model, and *p*(*x*) is the temperature integral, which has no analytical solution but can be approximated by empirical equations.

#### 3.4.1. Model-Free Approaches

The current study evaluated the kinetic parameters of rice residue pyrolysis using conversion techniques, including the Flynn–Wall–Ozawa (FWO) and Starink model-free isoconversional approaches. The FWO methodology, which builds upon Doyle’s enhancements [23], can be expressed as follows:

(7) $$\ln\beta =\ln\left(\frac{AE_{\alpha }}{RG\left(\alpha \right)}\right)-5.331-1.052\frac{E_{\alpha }}{RT}$$

The Starink method is shown in the following equation:

(8) $$\ln\left(\frac{\beta }{T^{1.92}}\right)=Const-1.0008\frac{E_{\alpha }}{RT}$$

The *E_a_* in the FWO method can be estimated on the basis of the slope of the line formed by plotting $\frac{1}{T}$ versus ln *β*. Furthermore, the *E_a_* for the Starink method is estimated on the basis of the slope of the $\ln\left(\frac{\beta }{T^{1.92}}\right)$ versus $\frac{1}{T}$ plot.

#### 3.4.2. Integral Master-Plot Method

The Tang–Liu–Zhang–Wang–Wang approximation of the empirical equation was employed to solve *p*(*x*), resulting in the following equation [22]:

(9) $$p\left(x\right)=\frac{exp\left(-x\right)}{x\times \left(\;1.0019882x+1.87391198\right)}$$

For the single-step degradation reaction, with *α* = 0.5 as the reference point, Equation (6) can be obtained:

(10) $$\frac{G\left(\alpha \right)}{G\left(0.5\right)}=\frac{p\left(x\right)}{p\left(x_{0.5}\right)}$$

where *G*(0.5) is the value at *α* = 0.5, and *x*_0_._5_ is the value of *x* at *α* = 0.5.

Table 6 provides an overview of the most commonly used reaction mechanism models. The best model is obtained by plotting the theoretical $\frac{G\left(\alpha \right)}{G\left(0.5\right)}$ and the experimental $\frac{p\left(x\right)}{p\left(x_{0.5}\right)}$ versus *x*. Equation (10) indicates that if the theoretical master plot $\frac{G\left(\alpha \right)}{G\left(0.5\right)}$ is equal to (or close to) the experimental master plot $\frac{p\left(x\right)}{p\left(x_{0.5}\right)}$, an appropriate reaction model has been selected.

### 3.5. Pyrolysis Performance Analysis

The biomass pyrolysis performance can be quantified on the basis of a number of characteristic parameters, including the initial devolatilization temperature (*T_i_*, °C), the peak temperature (*T_p_*, °C), the maximum rate of decomposition (−*R_p_*, %/min), the average rate of decomposition (−*R_v_*, %/min), the relative mass remaining at the end of the reaction (*M_f_*, %), and the comprehensive pyrolysis index (*CPI*, 10^−5^·%^3^·°C^−3^·min^−2^) [58], where *CPI* is given by the following equation:

(11) $$CPI=\frac{\left(-R_{p}\right)\times \left(-R_{v}\right)\times M_{\infty }}{T_{i}\times T_{p}\times ∆T_{1/2}}$$

where *M*_∞_ is the relative weight loss (*M*_∞_ = *M*_0_ − *M_f_*, where *M*_0_ is the initial devolatilized relative mass, %) and Δ*T*_1/2_ is the temperature interval (the pyrolysis temperature range when the decomposition rate is half of the maximum decomposition rate), with higher *CPI* values representing superior pyrolysis performance.

## 4. Conclusions

The low nitrogen and sulfur contents of RH and RS suggest their potential for use as clean biomass sources for energy and chemical applications. The best-fit models for the pseudo-components of RH were D1, A0.96, and A0.36, and those of RS were D2, A1, and A0.4. In terms of kinetic and thermodynamic properties, RS is more susceptible to thermal decomposition reactions than RH is. Compared with RH, RS results in a broader range of condensable by-products, with the highest abundance of C=O compounds. Our findings offer valuable insights into the optimization and upscaling of bioenergy production and the synthesis of high-value by-products through the controlled pyrolysis of rice residues.

## Figures and Tables

**Table 1:** Physicochemical analysis of RH and RS.

Sample		RH	RS
Ultimate analysis (wt%)	Carbon	37.73	41.67
	Nitrogen	0.47	0.64
	Hydrogen	4.50	5.94
	Sulphur	0.16	0.13
	Oxygen	32.58	35.40
Proximate analysis(wt%)	Moisture	7.25	7.56
	Volatiles	66.59	71.64
	Ash	14.82	11.66
	Fixed carbon	11.34	9.14
Component analysis(wt%)	Hemicellulose	36.71	47.27
	Cellulose	16.08	18.86
	Lignin	21.21	14.05
HHV (MJ/kg)		14.16	15.69

**Table 2:** The *E_a_* (kJ/mol) and *R*^2^ based on FWO and Starink for the three stages of the main RH and RS pyrolysis.

Samples	Conversion (α)	Stage 1				Stage 2				Stage 3			
		FWO		Starink		FWO		Starink		FWO		Starink	
		*E_a_*	*R* ^2^	*E_a_*	*R* ^2^	*E_a_*	*R* ^2^	*E_a_*	*R* ^2^	*E_a_*	*R* ^2^	*E_a_*	*R* ^2^
RH	0.1	200.3	0.9907	199.8	0.9900	172.7	0.9983	172.2	0.9981	188.4	0.9919	187.5	0.9910
	0.2	187.7	0.9967	187.1	0.9964	177.4	0.9985	177.0	0.9983	194.9	0.9929	192.5	0.9921
	0.3	181.8	0.9989	181.6	0.9988	182.3	0.9986	181.2	0.9984	204.4	0.9937	203.3	0.9930
	0.4	179.1	0.9997	178.6	0.9997	185.6	0.9986	185.4	0.9985	216.5	0.9946	214.9	0.9941
	0.5	177.1	0.9997	176.4	0.9997	189.4	0.9987	189.3	0.9986	230.5	0.9954	228.6	0.9950
	0.6	174.8	0.9995	174.7	0.9995	193.4	0.9986	192.6	0.9985	245.9	0.9955	244.6	0.9951
	0.7	173.0	0.9993	172.8	0.9992	195.2	0.9985	194.9	0.9984	265.1	0.9965	264.6	0.9963
	0.8	171.1	0.9990	170.7	0.9989	196.1	0.9985	195.7	0.9984	288.8	0.9987	287.6	0.9986
	0.9	168.6	0.9985	168.0	0.9984	196.4	0.9981	196.3	0.998	324.6	0.9985	324.4	0.9984
	Average	179.3		178.8		187.6		187.2		239.9		238.7	
RS	0.1	184.5	0.9475	178.7	0.9432	180.3	0.9892	180.3	0.9881	172.0	0.9980	170.4	0.9977
	0.2	177.3	0.9573	177.3	0.9533	181.2	0.9917	180.6	0.9909	174.1	0.9994	172.5	0.9993
	0.3	174.2	0.9626	175.6	0.9619	181.8	0.9913	181.2	0.9904	182.6	0.9997	178.7	0.9996
	0.4	174.2	0.9665	174.4	0.9632	182.0	0.9895	181.5	0.9883	193.5	0.9999	192.4	0.9999
	0.5	170.5	0.9691	170.4	0.9659	185.5	0.9927	185.3	0.9919	209.5	0.9999	209.1	0.9999
	0.6	167.6	0.9694	167.0	0.9662	186.3	0.9938	186.1	0.9931	217.4	0.9987	217.2	0.9985
	0.7	167.0	0.9677	166.5	0.9642	191.7	0.9934	191.6	0.9927	226.8	0.9985	226.9	0.9984
	0.8	166.2	0.9708	165.9	0.9676	192.1	0.9928	192.1	0.9920	228.3	0.9987	228.3	0.9985
	0.9	165.2	0.9711	165.4	0.9679	192.8	0.9936	192.7	0.9929	228.9	0.9999	228.6	0.9999
	Average	171.9		171.2		186.0		185.7		203.7		202.7	

**Table 3:** Kinetic triplets for the pseudo-components of RH and RS at five warming rates estimated with the master-plots.

Samples	Stage	*Β* (°C/min)	*E_a_* (kJ/mol)	*A* (s^−1^)	Reaction Model	R^2^
RH	Pseudo-hemicellulose	5	179.1	1.53 × 10^16^	1/(2*α*)	0.9987
		10		1.41 × 10^16^		0.9997
		20		1.42 × 10^16^		0.9999
		30		1.43 × 10^16^		0.9999
		50		1.34 × 10^16^		0.9997
	Pseudo-cellulose	5	187.4	6.27 × 10^15^	0.91(1 − *α*)[−ln(1 − *α*)]^−9/91^	0.9982
		10		6.45 × 10^15^		0.9992
		20		6.40 × 10^15^		0.9993
		30		6.57 × 10^15^		0.9988
		50		7.28 × 10^15^		0.9996
	Pseudo-lignin	5	239.3	1.53 × 10^17^	0.36(1 − *α*)[−ln(1 − *α*)]^−16/9^	0.9943
		10		1.88 × 10^17^		0.9977
		20		2.37 × 10^17^		0.9996
		30		2.41 × 10^17^		0.9995
		50		2.68 × 10^17^		0.9997
RS	Pseudo-hemicellulose	5	171.8	3.40 × 10^15^	[−ln(1 − *α*)]^−1^	0.9975
		10		2.71 × 10^15^		0.9998
		20		3.36 × 10^15^		0.9999
		30		2.55 × 10^15^		0.9995
		50		2.81 × 10^15^		0.9973
	Pseudo-cellulose	5	185.8	7.05 × 10^15^	1 − *α*	0.9948
		10		7.14 × 10^15^		0.9966
		20		7.61 × 10^15^		0.9940
		30		7.32 × 10^15^		0.9935
		50		7.94 × 10^15^		0.9914
	Pseudo-lignin	5	203.2	3.86 × 10^16^	0.4(1 − *α*)[−ln(1 − *α*)]^−3/2^	0.9997
		10		4.98 × 10^16^		0.9997
		20		6.25 × 10^16^		0.9978
		30		6.89 × 10^16^		0.9997
		50		7.38 × 10^16^		0.9995

**Table 4:** Pyrolysis characteristic parameters of RH and RS.

	*β* (°C/min)	*T_i_* (°C)	*T_p_* (°C)	*−R_p_* (%/min)	*−R_v_* (%/min)	*CPI* (10^−5^·%^3^·°C^−3^·min^−2^)
RH	5	144.8	328.9	3.84	0.41	3.42
	10	163.6	338.5	7.62	0.85	11.81
	20	171.6	349.5	14.81	1.72	40.56
	30	182.8	355.0	21.46	2.63	78.54
	50	191.1	362.3	35.18	4.42	198.46
RS	5	135.4	318.3	4.16	0.45	4.98
	10	145.9	328.7	8.07	0.91	16.48
	20	152.8	337.8	15.58	1.83	55.60
	30	159.7	343.8	22.85	2.78	109.17
	50	176.1	350.1	36.43	4.69	246.83

**Table 5:** Functional groups and typical gases from pyrolysis of RH and RS identified by TG-FTIR.

Wavelength (cm^−1^)	Functional Group	Product	Peak (cm^−1^)	
			RH	RS
4000–3450	O-H	H_2_O	3576	3591
3100–2650	C-H	CH_4_	2933	2932
2400–2250	C=O	CO_2_	2356	2357
2230–2020	C-O	CO	2183	2181
2000–1870	N-O	NO	1920	1921
1850–1640	C=O	Acids, aldehydes, ketones	1767	1744
1640–1570	N-O	NO_2_	1618	1621
1570–1480	C=C	Aromatics	1508	1508
1320–1000	C-O	Ether, Alcohol	1172	1163
750–600	C=O	CO_2_	669	668

**Table 6:** Differential and integral expressions for different reaction mechanism functions.

Mechanism	*f*(*α*)	*G*(*α*)
Diffusion models		
1-D diffusion (D1)	1/(2*α*)	*α* ^2^
2-D diffusion (D2)	[−ln(1 − *α*)]^−1^	(1 − *α*)ln(1 − *α*) + *α*
3-D diffusion (D3)	[(3/2) (1 − *α*)^2/3^]/[1 − (1 − *α*)^1/3^]	[1 − (1 − *α*)^1/3^]2
4-D diffusion (D4)	[(3/2) (1 − *α*)^1/3^]/[1 − (1 − *α*)^1/3^]	(1 − 2*α*/3) − (1 − *α*)^2/3^
Geometrical contraction models		
Contracting area (R2)	2 (1 − *α*)1/2	1 − (1 − *α*)1/2
Contracting volume (R3)	3 (1 − *α*)1/2	1 − (1 − *α*)1/3
Reaction order models		
First-order (F1)	1 − *α*	−ln(1 − *α*)
Second-order (F2)	(1 − *α*)^2^	(1 − *α*)^−1^ − 1
Third-order (F3)	(1 − *α*)^3^	[(1 − *α*)^−2^ − 1]/2
Nucleation models		
Avarami–Erofeev (A1)	1 − *α*	−ln(1 − *α*)
Avarami–Erofeev (A2)	2 (1 − *α*)[−ln(1 − *α*)]^1/2^	[−ln(1 − *α*)]^1/2^
Avarami–Erofeev (A3)	3 (1 − *α*)[−ln(1 − *α*)]^2/3^	[−ln(1 − *α*)]^1/3^
Avarami–Erofeev (A4)	4 (1 − *α*)[−ln(1 − *α*)]^3/4^	[−ln(1 − *α*)]^1/4^
Avarami–Erofeev (A*n*)	*n* (1 − *α*)[−ln(1 − *α*)]^(*n*−1)/*n*^	[−ln(1 − *α*)]^1/*n*^
Power law models		
2-power law (P2)	2*α*^1/2^	*α* ^1/2^
3-power law (P3)	3*α*^1/3^	*α* ^1/3^
4-power law (P4)	4*α*^1/4^	*α* ^1/4^

## Data Availability

The data attained during the current study are available from the corresponding author on reasonable request.

## Supplementary Materials

The following supporting information can be downloaded at: https://www.mdpi.com/article/10.3390/molecules30010010/s1, Table S1: By-products of pyrolysis of RH and RS identified by Py-GC/MS; Figure S1: Fourier infrared spectra of RH and RS; Figure S2: *P* (*x*)/*P* (*x*_0.5_) versus conversion degree for the five heating rates and the sub-stages of the pyrolysis: for (a–c) RH and (d–f) RS. Figure S3: 3D FTIR plot for the pyrolysis of RH and RS.
