# Supplementary material for: Analysis of the Pyrolysis Kinetics, Reaction Mechanisms, and By-Products of Rice Husk and Rice Straw via TG-FTIR and Py-GC/MS
Source: Molecules. 2024 Dec 24;30(1):10. doi: 10.3390/molecules30010010 (PMC11721799; doi:10.3390/molecules30010010)
Supplement: Supplementary file 1 [file molecules-30-00010-s001.zip › molecules-3343480-supplementary.pdf]

Table S1

By-products of pyrolysis of RH and RS identified by Py-GC/MS.

No	Name of compound	Chemical formula	MW	Group	Area (%)	
					RH	RS
1	1-Propanol, 2-amino-, (S)-	C_3H_9NO	75	Nitrides	1.97	2.24
2	Acetaldehyde	C_2H_4O	44	Aldehydes	1.97	1.31
3	Furan	C_4H_4O	68	Furans	0.93	0.66
4	Methyl glyoxal	$C_3H_4O_2$	72	Aldehydes	3.46	3.00
5	2-Butenal	C_4H_6O	70	Aldehydes	0.26	0.28
6	Furan, 2-methyl-	C_5H_6O	82	Furans	0.42	0.81
7	Furan, 3-methyl-	C_5H_6O	82	Furans	0.13	0.00
8	2,3-Butanedione	$C_4H_6O_2$	86	Ketones	2.55	2.01
9	2-Buten-1-ol	C_4H_8O	72	Alcohols	0.00	0.37
10	1-Propanol	C_3H_8O	60	Alcohols	4.81	3.66
11	Aminoacethydrazide	$C_2H_7N_3O$	89	Nitrides	0.00	0.75
12	2,2'-Bioxirane	$C_4H_6O_2$	86	Ethers	0.13	0.00
13	Ethanol	C_2H_6O	46	Alcohols	0.18	0.00
14	Furan, 2,5-dimethyl-	C_6H_8O	96	Furans	0.18	0.33
15	2-Hexanol	$C_6H_{14}O$	102	Alcohols	0.00	0.74
16	2-Butenal, (E)-	C_4H_6O	70	Aldehydes	0.79	0.00
17	Acetic acid	$C_2H_4O_2$	60	Acids	12.08	7.91
18	Heptane	C_7H_{16}	100	Hydrocarbons	0.00	0.69
19	2-Propanone, 1-hydroxy-	$C_3H_6O_2$	74	Ketones	5.60	3.90
20	Toluene	C_7H_8	92	Hydrocarbons	0.21	0.00
21	Carbohydrazide	CH_6N_4O	90	Nitrides	0.20	0.46
22	1,4-Butanediamine	$C_4H_{12}N_2$	88	Nitrides	0.41	0.54
23	Propanoic acid	$C_3H_6O_2$	74	Acids	0.54	0.56
24	3-Butyn-2-ol	C_4H_6O	70	Alcohols	0.00	0.17
25	1-Hydroxy-2-butanone	$C_4H_8O_2$	88	Ketones	2.72	2.01
26	Pyrrole	C_4H_5N	67	Nitrides	0.40	0.49
27	2(3H)-Furanone	$C_4H_4O_2$	84	Furans	0.00	0.19
28	2-Ethylideneamino-propionitrile	$C_5H_8N_2$	96	Nitrides	0.00	0.64
29	Carbonocyanidic acid, ethyl ester	$C_4H_5NO_2$	99	Nitrides	0.33	0.00
30	3-Buten-2-one, 3-methyl-	C_5H_8O	84	Ketones	0.25	0.70
31	2-Hydroxy-gamma-butyrolactone	$C_4H_6O_3$	102	Esters	3.22	2.73
32	5-Heptyn-3-ol	$C_7H_{12}O$	112	Alcohols	0.00	0.12
33	1,5-Hexadien-3-yne	C_6H_6	78	Hydrocarbons	0.67	0.00
34	2(1H)-Pyridinone	C_5H_5NO	95	Nitrides	1.59	1.17
35	2-Cyclopenten-1-one	C_5H_6O	82	Ketones	1.25	0.93
36	1-Hexene, 4,5-dimethyl-	C_8H_{16}	112	Hydrocarbons	0.00	0.18
37	3-Furanmethanol	$C_5H_6O_2$	98	Furans	1.55	3.73
38	2-Propanone, 1-(acetyloxy)-	$C_5H_8O_3$	116	Ketones	1.08	1.08
39	2-Butanone	C_4H_8O	72	Ketones	0.77	0.84
40	2-Cyclopenten-1-one, 2-methyl-	C_6H_8O	96	Ketones	0.42	0.47
41	Ethanone, 1-(2-furanyl)-	$C_6H_6O_2$	110	Furans	0.31	0.39
42	2-Propenoic acid, 2-methyl-, 2-propynyl ester	$C_6H_{10}O_3$	130	Esters	0.00	0.25
43	Cyclopent-4-ene-1,3-dione	$C_5H_8O_2$	100	Alcohols	0.12	0.17
44	D-Limonene	$C_{10}H_{16}$	136	Ketones	0.22	0.13
45	2-Butanamine, N-(1-methylpropyl)-	$C_8H_{19}N$	129	Nitrides	0.00	0.16
46	1,2-Cyclopentanedione	$C_5H_6O_2$	98	Ketones	2.54	3.04
47	Cyclopentanone, 2-methyl-	$C_6H_{10}O$	98	Ketones	0.17	0.00
48	2,5-Hexanedione	$C_6H_{10}O_2$	114	Ketones	0.13	0.25
49	1-Heptene	C_7H_{14}	98	Hydrocarbons	0.00	0.21
50	2-Furanmethanol	$C_5H_6O_2$	98	Furans	0.23	0.59
51	2-Butanone, 3,3-dimethyl-	$C_6H_{12}O$	100	Ketones	0.19	0.25
52	2-Furancarboxaldehyde, 5-methyl-	$C_5H_4O_2$	96	Furans	0.47	0.23
53	Furan, 2,3,5-trimethyl-	$C_7H_{10}O$	110	Furans	0.00	0.25
54	Butyrolactone	$C_4H_6O_2$	86	Esters	0.95	1.29
55	2(5H)-Furanone	$C_4H_4O_2$	84	Furans	0.87	1.01
56	2-Pentanone, 3-methylene-	$C_6H_{10}O$	98	Ketones	0.00	0.18
57	Butanediamide, 2-methylene-	$C_5H_8N_2O_2$	128	Nitrides	0.31	0.31
58	1H-1,2,4-Triazole, 3-nitro-	$C_2H_2N_4O_2$	14	Nitrides	0.00	0.15
59	2(5H)-Furanone, 3-methyl-	$C_5H_6O_2$	98	Furans	0.17	0.30
60	1,2-Cyclopentanedione, 3-methyl-	$C_5H_6O_2$	98	Ketones	2.47	2.35
61	2-Furanone, 2,5-dihydro-3,5-dimethyl	$C_6H_8O_2$	112	Esters	0.17	0.25
62	2-Cyclopenten-1-one, 2-hydroxy-3,4-dimethyl-	$C_5H_6O_2$	98	Ketones	0.14	0.15
63	Phenol	C_6H_6O	94	Phenols	2.09	1.35
64	Phenol, 2-methoxy	$C_7H_8O_2$	124	Phenols	2.50	1.03
65	2-Cyclopenten-1-one, 3-ethyl-	$C_7H_{10}O$	110	Ketones	0.14	0.35
66	Phenol, 2-methyl-	C_7H_8O	108	Phenols	0.31	0.30
67	Furyl hydroxymethyl ketone	$C_6H_6O_3$	126	Furans	0.00	0.19
68	1,1'-Bicyclopentyl	$C_{10}H_{18}$	138	Hydrocarbons	0.00	0.12
69	Propanoic acid, ethenyl ester	$C_5H_8O_2$	100	Esters	0.47	0.91
70	3-Penten-2-one, 3-ethyl-4-methyl-	$C_8H_{14}O$	126	Ketones	0.49	0.63
71	Maltol	$C_6H_6O_3$	126	Ketones	0.16	0.32
72	2H-Pyran-2-one, tetrahydro-	$C_5H_8O_2$	100	Esters	0.19	0.39
73	2-Hydroxy-3,5-diethyl-5-methylcyclopent-2-en-1-one	$C_{10}H_{16}O_2$	168	Ketones	0.00	0.15
74	p-Cresol	C_7H_8O	108	Phenols	0.84	0.74

75	2-Pyrrolidinone	C_4H_7NO	85	Nitrides	0.18	0.34
76	1-Butanol, 2,2-dimethyl-	$C_6H_{14}O$	102	Alcohols	0.00	0.33
77	Succinic anhydride	$C_4H_4O_3$	100	Esters	0.14	0.17
78	3-Heptyn-1-ol	$C_7H_{12}O$	112	Alcohols	0.00	0.13
79	1-Nonyne	C_9H_{16}	124	Hydrocarbons	0.00	0.22
80	3-Buten-2-ol	C_4H_8O	72	Alcohols	3.87	3.18
81	Phenol, 2-ethyl-	$C_8H_{10}O$	122	Phenols	0.16	0.22
82	2-Acetyl-5-methylfuran	$C_7H_8O_2$	124	Furans	0.00	0.17
83	2-Hydroxy-3-propyl-2-cyclopenten-1-one	$C_8H_{12}O_2$	140	Ketones	0.00	0.16
84	Phenol, 4-ethyl-	$C_8H_{10}O$	122	Phenols	0.67	0.55
85	2H-Pyran-3(4H)-one, dihydro-6-methyl-	$C_5H_8O_2$	100	Ketones	0.28	0.26
86	3-Hexene, (Z)-	C_6H_{12}	84	Hydrocarbons	0.32	0.45
87	Phenol, 4-ethyl-2-methoxy-	$C_9H_{12}O_2$	152	Phenols	0.58	0.26
88	1,4-Dioxaspiro[2.4]heptan-5-one, 6-methyl-	$C_5H_6O_3$	114	Esters	0.00	0.21
89	Phenol, 2-ethyl-5-methyl-	$C_9H_{12}O$	136	Phenols	0.24	0.33
90	3,4-Anhydro-d-galactosan	$C_6H_8O_4$	144	Acidsugars	0.12	0.30
91	2,3-Anhydro-d-mannosan	$C_6H_8O_4$	144	Acidsugars	0.14	0.45
92	Ethanone, 1-(2,4-dihydroxyphenyl)-	$C_8H_8O_3$	152	Phenols	0.00	0.17
93	1,4:3,6-Dianhydro-.alpha.-d-glucopyranose	$C_6H_8O_4$	144	Acidsugars	0.39	0.67
94	Phenylcyclopentylamine	$C_{11}H_{15}N$	161	Nitrides	0.18	0.00
95	1-Penten-3-ol	C_5H_{10}	86	Alcohols	0.00	0.31
96	Furan, 2-(1,1-dimethylethyl)-4-methyl-	$C_8H_{16}O_2$	144	Ethers	0.00	0.11
97	Benzaldehyde, 3-methyl-	C_8H_8O	120	Aldehydes	4.94	2.91
98	2-Methoxy-4-vinylphenol	$C_9H_{10}O_2$	150	Phenols	2.55	1.86
99	2-Oxepanone, 7-butyl-	$C_{10}H_{18}O_2$	170	Esters	0.00	0.14
100	5-Hydroxymethylfurfural	$C_6H_6O_3$	126	Furans	0.25	0.69
101	Catechol	$C_6H_6O_2$	110	Phenols	0.00	0.20
102	5-Hydroxymethyldihydrofuran-2-one	$C_5H_8O_3$	116	Esters	0.45	0.91
103	2,6-Dimethoxyphenol	$C_8H_{10}O_3$	154	Phenols	2.09	1.74
104	2(3H)-Furanone, dihydro-4-hydroxy-	$C_4H_6O_2$	86	Esters	0.00	0.21
105	3-Hexanone	$C_6H_{12}O$	100	Ketones	0.13	0.17
106	Succinic acid, 2,4-dimethylpent-3-yl ethyl ester	$C_{13}H_{24}O_4$	224	Esters	0.19	0.00
107	Digitoxose	$C_6H_{12}O_4$	148	Acidsugars	0.16	0.21
108	Eugenol	$C_{10}H_{12}O_2$	164	Phenols	0.59	0.13
109	1,2,3-Trimethoxybenzene	$C_9H_{12}O_3$	168	Ethers	0.88	0.86
110	Vanillin	$C_8H_8O_3$	152	Phenols	0.33	0.21
111	Benzaldehyde, 4-hydroxy-	$C_7H_6O_2$	122	Phenols	0.15	0.15
112	1,4-Benzenediol, 2-methyl-	$C_7H_8O_3$	140	Phenols	0.12	0.35
113	2-Butene-1,4-diol	$C_4H_8O_2$	88	Alcohols	0.18	0.57
114	Ethanone, 1-(2,6-dihydroxy-4-methoxyphenyl)-	$C_9H_{10}O_4$	182	Phenols	0.24	0.22
115	Butane, 1-(1-ethoxyethoxy)-	$C_8H_{18}O_2$	146	Ethers	0.25	0.47
116	2-Butenoic acid, butyl ester	$C_8H_{14}O_2$	142	Esters	0.00	0.40
117	2-Acetoxydodecane	$C_{14}H_{28}O_2$	228	Esters	0.00	0.43
118	Benzaldehyde, 3-hydroxy-	$C_7H_6O_2$	122	Phenols	0.24	0.25
119	Acetic acid, octyl ester	$C_{10}H_{20}O_2$	172	Esters	0.00	0.13
120	4-Methyl-2,5-dimethoxybenzaldehyde	$C_{12}H_{19}NO_2$	209	Nitrides	0.60	0.44
121	2-Propanone, 1-(4-hydroxy-3-methoxyphenyl)-	$C_{10}H_{12}O_3$	180	Phenols	0.42	0.12
122	2-Hydroxy-4-isopropyl-7-methoxytropone	$C_{10}H_{12}O_2$	164	Ketones	0.00	0.14
123	2H-1-Benzopyran-2-one, 3,4-dihydro-6-hydroxy-	$C_{14}H_{14}O_3$	230	Phenols	0.20	0.25
124	3-Acetonyl-2-hydroxy-2,4,6-cycloheptatrien-1-one	$C_{10}H_{10}O_3$	178	Esters	0.00	0.39
125	2,6-Di-n-propyl-4-phenylpyridine	$C_{17}H_{21}N$	239	Nitrides	0.14	0.40
126	β-D-Glucopyranose, 1,6-anhydro-	$C_6H_{10}O_5$	162	Acidsugars	1.63	4.53
127	Hexadecenenitrile	$C_{16}H_{29}N$	235	Nitrides	0.00	0.17
128	(E)-2,6-Dimethoxy-4-(prop-1-en-1-yl)phenol	$C_{11}H_{14}O_3$	194	Phenols	0.40	0.38
129	1,6:2,3-Dianhydro-4-O-acetyl-β-d-talopyranose	$C_8H_{10}O_5$	186	Acidsugars	0.00	0.16
130	Ethanone, 1-(4-hydroxy-3,5-dimethoxyphenyl)-	$C_{11}H_{12}O_2$	176	Phenols	0.00	0.21
131	Desaspidinol	$C_{11}H_{14}O_4$	210	Phenols	0.12	0.15
132	Hexadecanoic acid	$C_{16}H_{32}O_2$	256	Acids	2.46	1.32
133	Heptadecanoic acid, 10-methyl-, methyl ester	$C_{19}H_{38}O_2$	298	Esters	0.12	0.00
134	1-(3-Hydroxy-4-methylphenyl)-1,3,3,6-tetramethylindan-5-ol	$C_{20}H_{24}O_2$	296	Phenols	0.20	0.00
135	Tridecane, 3-methylene-	$C_{14}H_{28}$	196	Hydrocarbons	0.00	0.17
136	1-Dodecanol, 2-octyl-	$C_{20}H_{42}O$	298	Alcohols	0.00	0.12
137	Octadecanoic acid	$C_{18}H_{36}O_2$	284	Acids	0.00	0.75
138	acetonitrile, 2-(2,6-diphenyl-4H-pyran-4-ylidene)-	$C_{19}H_{13}NO$	271	Nitrides	0.84	0.00
139	Tetracosane	$C_{24}H_{50}$	338	Hydrocarbons	0.00	0.96
140	3-Tetradecanol	$C_{14}H_{30}O$	214	Alcohols	0.31	0.13
141	Cyclohexane, ethyl-	C_8H_{16}	112	Hydrocarbons	0.14	0.00
142	Fumaric acid, 2-ethoxyethyl octyl ester	$C_{16}H_{28}O_5$	300	Esters	0.62	0.20
143	2-Hexadecanone	$C_{16}H_{32}O$	240	Ketones	0.53	0.20
144	Ethanol, 2-(octadecyloxy)-	$C_{20}H_{42}O_2$	314	Alcohols	0.40	0.68
145	Hexacosane	$C_{26}H_{54}$	366	Hydrocarbons	0.21	0.00
146	Bis(2-ethylhexyl) phthalate	$C_{24}H_{38}O_4$	390	Esters	0.61	0.25
147	Heptacosane	$C_{27}H_{56}$	380	Hydrocarbons	0.21	0.00
148	Hexatriacontane	$C_{36}H_{74}$	506	Hydrocarbons	0.00	0.50
149	Squalene	$C_{30}H_{50}$	410	Hydrocarbons	0.70	0.16
150	Supraene	$C_{30}H_{50}$	410	Hydrocarbons	0.42	0.11
151	Triacontane, 1-bromo-	$C_{44}H_{90}$	618	Hydrocarbons	0.00	0.81

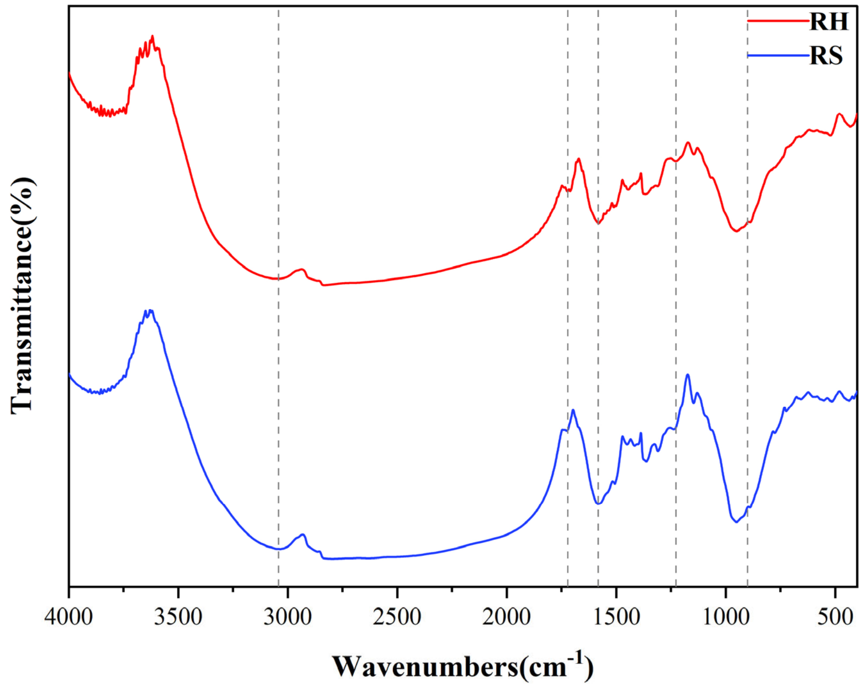

Figure S1. Fourier infrared spectra of RH and RS.

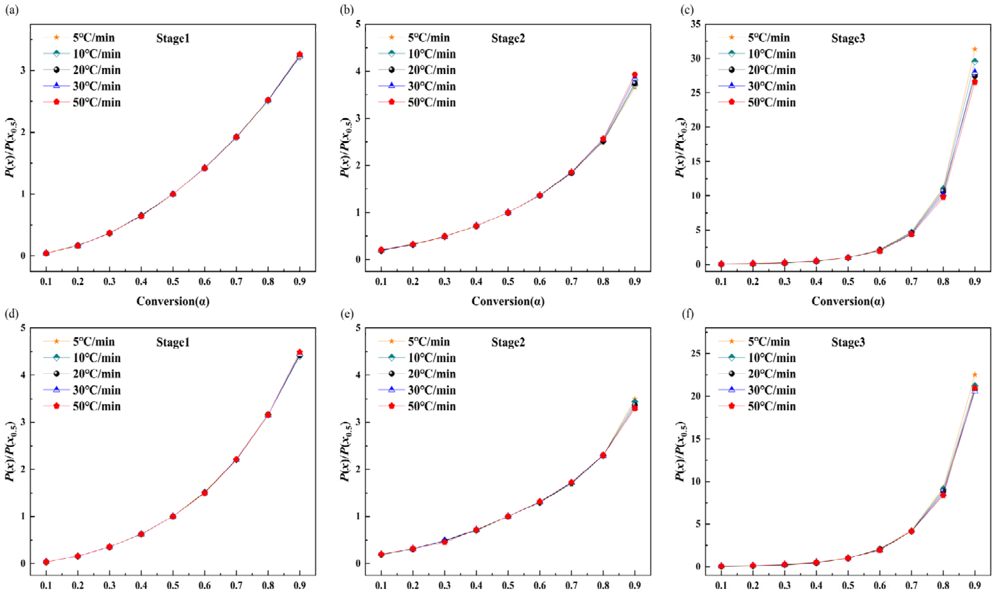

Figure S2. $P(x)/P(x_{0.5})$ versus conversion degree for the five heating rates and the sub-stages of the pyrolysis: for (a-c) RH and (d-f) RS.

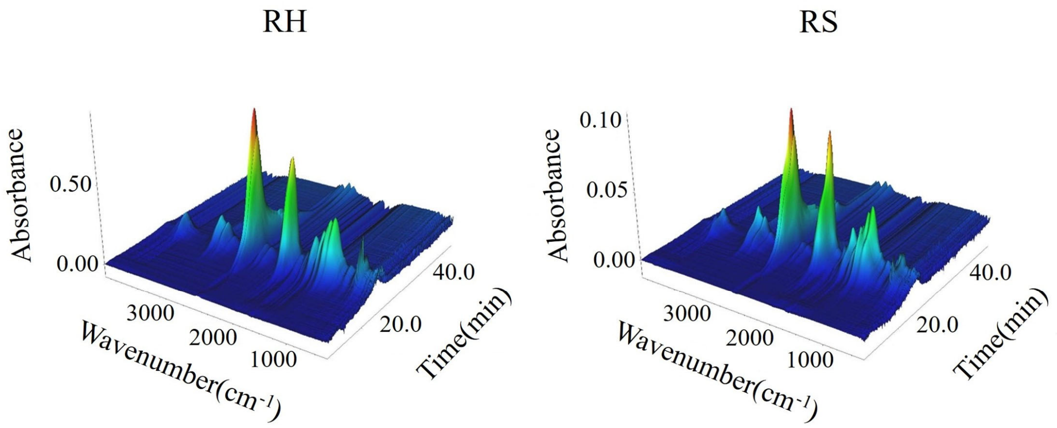

Figure S3. 3D FTIR plot for the pyrolysis of RH and RS.
